# Application of UAV Multisensor Data and Ensemble Approach for High-Throughput Estimation of Maize Phenotyping Traits

**DOI:** 10.34133/2022/9802585

**Published:** 2022-08-27

**Authors:** Meiyan Shu, Shuaipeng Fei, Bingyu Zhang, Xiaohong Yang, Yan Guo, Baoguo Li, Yuntao Ma

**Affiliations:** ^1^College of Land Science and Technology, China Agricultural University, Beijing 100091, China; ^2^State Key Laboratory of Plant Physiology and Biochemistry, National Maize Improvement Center of China, China Agricultural University, Beijing 100091, China

## Abstract

High-throughput estimation of phenotypic traits from UAV (unmanned aerial vehicle) images is helpful to improve the screening efficiency of breeding maize. Accurately estimating phenotyping traits of breeding maize at plot scale helps to promote gene mining for specific traits and provides a guarantee for accelerating the breeding of superior varieties. Constructing an efficient and accurate estimation model is the key to the application of UAV-based multiple sensors data. This study aims to apply the ensemble learning model to improve the feasibility and accuracy of estimating maize phenotypic traits using UAV-based red-green-blue (RGB) and multispectral sensors. The UAV images of four growth stages were obtained, respectively. The reflectance of visible light bands, canopy coverage, plant height (PH), and texture information were extracted from RGB images, and the vegetation indices were calculated from multispectral images. We compared and analyzed the estimation accuracy of single-type feature and multiple features for LAI (leaf area index), fresh weight (FW), and dry weight (DW) of maize. The basic models included ridge regression (RR), support vector machine (SVM), random forest (RF), Gaussian process (GP), and K-neighbor network (K-NN). The ensemble learning models included stacking and Bayesian model averaging (BMA). The results showed that the ensemble learning model improved the accuracy and stability of maize phenotypic traits estimation. Among the features extracted from UAV RGB images, the highest accuracy was obtained by the combination of spectrum, structure, and texture features. The model had the best accuracy constructed using all features of two sensors. The estimation accuracies of ensemble learning models, including stacking and BMA, were higher than those of the basic models. The coefficient of determination (*R*^2^) of the optimal validation results were 0.852, 0.888, and 0.929 for LAI, FW, and DW, respectively. Therefore, the combination of UAV-based multisource data and ensemble learning model could accurately estimate phenotyping traits of breeding maize at plot scale.

## 1. Introduction

Leaf area index (LAI) is one of key traits of characterizing crop growth, which is highly relevant to crop photosynthesis and transpiration [1–3]. Aboveground biomass (AGB) is an important basis for crop yield formation [4, 5]. Therefore, accurate and rapid estimation of maize LAI and AGB is helpful for high-throughput screening of breeding maize.

The manual measurement of crop phenotypic traits is intensive in terms of both labor and time [6–8]. Moreover, destructive sampling of a large area in the field will affect crop growth. In recent years, unmanned aerial vehicle (UAV) imaging technology provides an effective means to obtain crop phenotypic traits at plot scale [9, 10]. UAV imaging technology has been widely used to research of phenotypic trait estimation for crop breeding, including emergence rate [11], LAI [12, 13], plant height [14], biomass [15], and lodging [16].

Many research findings revealed that the spectrum, structure, texture, temperature, and other information extracted from UAV images can be used for estimating crop phenotypic traits [17, 18]. Spectrum, structure and texture information have been widely used in estimating crop LAI, above ground biomass, yield, nitrogen content, and chlorophyll content [12, 13, 19, 20]. The fusion of multisource data can complement each other to improve the accuracy of estimating crop phenotypic traits [21]. For example, the combination of structure and spectrum can effectively solve the problem of spectrum saturation at later crop growth stage [22–24]. The potential of multisource data fusion in estimating phenotypic traits of different breeding maize materials need to be further explored.

Machine learning methods can estimate crop phenotypic traits with high accuracy [25–27], which have strong ability to solve nonlinear problems and flexibility of integrating multisource data [28–30]. Commonly used machine learning algorithms include such as support vector machines (SVM), random forests (RF), and artificial neural networks (ANN). However, these methods are prone to overfitting in the case of limited training samples [10]. Ensemble learning is an extension of machine learning and can improve the generalization ability by integrating the output results of each base model through secondary learning methods [30, 31]. There are three common ensemble learning methods, including bagging, boosting, and stacking [32, 33]. The ensemble methods of bagging and boosting can perform secondary learning by assigning higher weights to the samples with poor training effect, which improves the model prediction accuracy and generalization ability [34, 35]. However, these two methods can only integrate the same type of decision tree models, and have difficulty with integrating the advantages of different types of models. Stacking is a hierarchical model integration framework. Firstly, different types of basic models are used to train the dataset. Secondly, the training results obtained by each basic model are formed into a new training set as the input of the second learning to make the final decision [36, 37]. Because outputs are derived from multiple basic models, the stacking ensemble learning can increase accuracy, robustness, and overall generalization of the estimation model [32, 33, 38]. At present, there has been limited research on phenotypic traits for breeding maize materials using UAV-based multisource data and ensemble learning model. In the reported studies, various machine learning including deep learning methods have been proposed to fuse multisource image data for assessing crop traits. These models have achieved good accuracy on specific crops in specific areas, but it is difficult to prove the universality of these models. Through two phases of learning, ensemble models may have the potential to unify the result from different models, which are more beneficial than traditional machine learning methods.

Due to the uncertainty of model parameter and structure, Bayesian Model Averaging (BMA) takes the posterior probability of each basic models as weights in the secondary learning to obtain a more reliable probability distribution of predictive variables [39, 40] BMA is considered the most popular modeling method for avoiding the uncertainty in the modeling process, which can produce more reliable and accurate prediction results. At present, BMA has been widely used in various fields [41–43].

The primary objective of this study was to use UAV-based digital and multispectral data for estimating phenotypic traits of breeding maize materials across all growth stages by ensemble learning method. Specific objectives were as follows: (1) test the application potential of spectrum, texture, and structure information and their combinations in estimating maize phenotypic traits, such as LAI, FW, and DW; (2) compare the performances of five basic models of machine learning and two ensemble models; and (3) evaluate if data fusion and ensemble learning can improve the accuracy and stability of estimating phenotypic traits for breeding maize materials.

## 2. Material and Methods

### 2.1. Study Area and Experimental Setup

**Figure 1:**
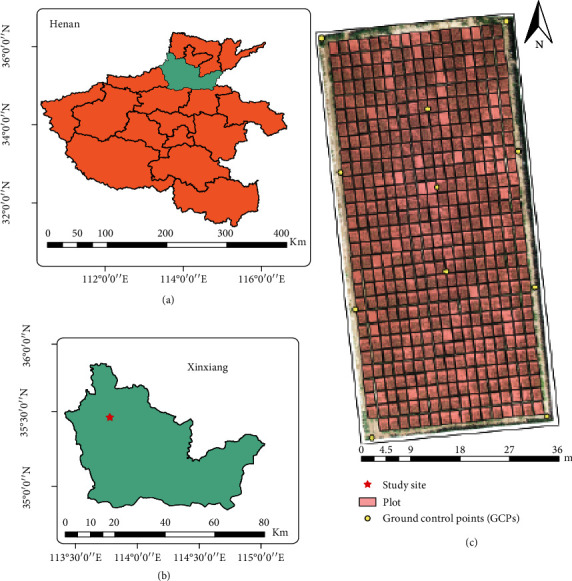
Location of the experimental site.

The experimental site lied in the Xinxiang County, Henan Province, China (113°51′ E, 35°18′ N) (Figure 1).

Xinxiang County belongs to warm temperate continental monsoon climate zone. The average annual temperature is 14°C in year 2020. The average precipitations are about 550 mm in year 2020 with the wettest months in July and August. Due to the flat terrain and the fertile soil, the maize yield in Xinxiang County is generally very high.

The sowed maize inbred line had extensive genetic diversity, which included 483 varieties used in the experiment. The sowing dates were June 23, 2020. Each genotypic material was sowed on a plot. Zheng58 was used as reference material and sowed every 50 plant lines. There were 492 plots in total. The width of each plot was 1.2 m, while the length was 2.5 m. The row spacing of each plot was 0.6 m, while the plant spacing was 0.25 m. The fertilization and irrigation modes in each plot were the same and consistent with the local conventional modes.

### 2.2. Data Acquisition

#### 2.2.1. Sample Data Collection

According to genetic diversity estimation, we selected 55 plots as samples for measuring the phenotypic traits, including dry weight (DW), plant height (PH), LAI, and fresh weight (FW). The growth of maize plants in the sampling plots was relatively uniform. In order not to affect the grain yield measurement in the harvest stage, one plant representing the average growth in each plot was selected in each observation stage. The measuring dates include July 20, July 30, August 18, and September 18, 2020, corresponding to the day after sowing (DAS) = 27, DAS = 37, DAS = 56, and DAS = 87, respectively. Detailed information on PH measurement is found in the study of Shu et al. [2]. We cut off the maize plant from the root. The LAI was calculated by the maximum width and length of each leaf according to the method of Montgomery [44]. The stem, leaves, and ears of the sampling plant were separated and measured their FW, respectively. Then the organs of the sample plant were put in envelopes, respectively, and dried to constant weight. The total FW and DW (g/m^2^) of the sampling plot was calculated by the planting density and the FW and DW of sample plant. Due to the inconsistency of seedling emergence rate in each observation stage, the planting density was determined by number of actual plants per plot.

#### 2.2.2. UAV Imaging

The UAV-based RGB and multispectral images were obtained on the same day of field observation. Before imaging, we evenly arranged 11 ground control points (GCPs) (Figure 1), and fixed their position with the RTK (CHCNAV - T8, Shanghai, China).

The UAV-based RGB data were obtained using by DJI Phantom 4 Pro v2.0 (DJI, Shenzhen, China) in this study. The duration of UAV is around 30 minutes. The imaging sensor is 20 megapixels with the RGB image resolution of 5472∗3648. The altitude was set to 30 m. The overlap ratio of images was 80%. The stitching of RGB images was carried out in Agisoft PhotoScan Professional (Agisoft LLC, St. Petersburg, Russia). During image splicing, 11 GCPs were used for geometric correction. Finally, we acquired the digital surface model (DSM) and digital orthophoto model (DOM) of the experimental site.

**Figure 2:**
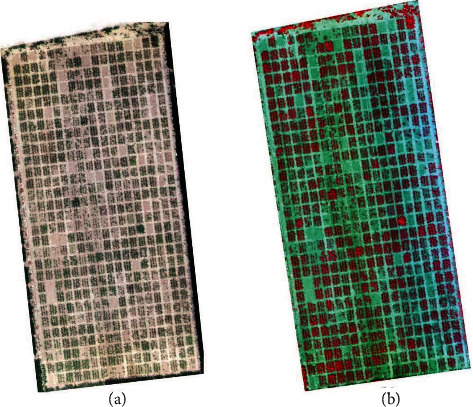
RGB (a) and multispectral (b) images of UAV acquired on July 30, 2020. (b) The band combination is nir band, red band, and green band.

The multispectral images were acquired using by the Parrot Sequoia imaging system (MicaSense Inc., Seattle, USA). The Sequoia sensor can obtain four multispectral bands, including near-infrared, red edge, and red and green bands. Different bands have different bandwidths. Among the four bands, the bandwidth of red-edge band is 10 nm, and the other three are all 40 nm. The imaging system contains the sunshine sensor. During the flight, the multispectral images can be automatically calibrated by the sunshine sensor with the change of light [45]. The flight height and overlap rate of UAV-based multispectral images were the same as the UAV-based RGB images. Radiometric calibration was performed using standard whiteboard images of four bands which were acquired before the flight. The stitching of multispectral images was carried out in the Pix4Dmapper (PIX4D, Lausanne, Switzerland). Similar to the stitching process of RGB images, 11 GCPs were used for geometric correction.  Figure 2 shows the RGB (a) and multispectral (b) images of UAV acquired on July 30, 2020.

### 2.3. Feature Extraction

**Figure 3:**
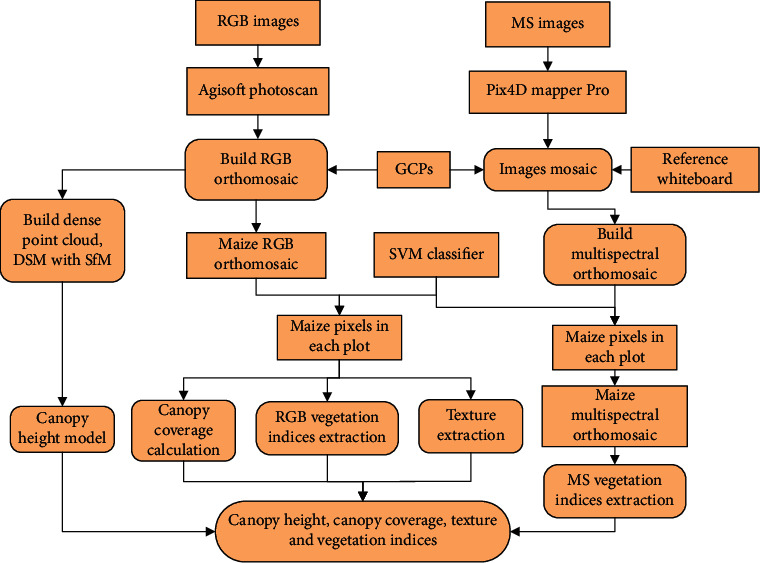
Processing workflow for UAV-based feature variables. DSM is the digital surface model. SfM is the structure from motion. MS and GCPs are the multispectral and ground control points.

Compared with multispectral images, the RGB images obtained at the same flight height have higher spatial resolution and are more useful for texture information extraction. In this study, RGB images were used to obtain canopy coverage, PH, and texture information of each plot. The DN value of RGB images is less sensitive to the changes of light intensity. Studies showed that the spectral indices calculated based on the DN value of RGB images could be used to estimate crop phenotypic traits [12, 13]. Therefore, a series of spectral vegetation indices were calculated using the DN value of RGB images and the reflectance of multispectral images to estimate LAI, FW, and DW of maize plants. The extraction process of UAV-based feature variables is shown in Figure 3.

#### 2.3.1. Canopy Coverage

Canopy coverage represents the proportion of crop canopy vertical projection area to ground area [7, 8, 46]. Canopy coverage can reflect the growth status of crops [2, 7, 8]. As the spatial resolution of RGB image was higher than that of the multispectral image, the canopy coverage of each plot was extracted based on the RGB image. In this study, we used the SVM classifier to extract maize pixels for calculating the canopy coverage of each plot [47]. SVM classifier was obtained by calling scikit-learn library based on Python 3.6. The pixels of RGB image of each sample plot at each growing stage was classified into maize, soil, shadow, and others. The vector files obtained in ArcGIS 10.6 (ESRI, Redlands, USA) and SVM classifier were used to segment the images, extract maize plants, and calculate the canopy coverage of each plot in Python 3.6. The RGB images containing only maize plants were obtained through the mask.

#### 2.3.2. Plant Height Estimation

PH is an important parameter to describe the crop growth status, which is proportional to dry weight of maize plant and is highly relevant to above-ground biomass and grain yield [48, 49]. Therefore, PH was used as an independent variable to participate in the model construction of LAI, FW, and DW. The difference between DSM and DEM can be used to estimate the crop PH [50]. The detailed process of plant height estimation was referred to the study of Shu et al. [2].

#### 2.3.3. Texture Information

Texture information is a common visual phenomenon. The texture information can quantify the attributes of surface structure and organization arrangement. Gray-level cooccurrence matrix (GLCM) is a widely used method to extract texture information [12, 13], which reflects the information of direction, distance, and gray changes of the image. The RGB image only including maize plants was transformed into the gray image. Then the texture information of each plot was extracted, and the specific parameters included mean, variance, contrast, energy, entropy, homogeneity, autocorrelation, dissimilarity, and correlation. After many attempts, the size of the sliding window was set as 7 × 7, and the sliding step was set as 2.

#### 2.3.4. Vegetation Indices

The same as the RGB images processing method, we obtained the multispectral images containing only maize plants. The reflectance of each band of maize canopy in each plot was extracted from RGB images and multispectral images. In the research of crop growth, it is a common method to estimate crop phenotypic traits using vegetation indices constructed by specific bands as independent variables. These vegetation indices with certain physical significance not only enhance a certain signal of vegetation, but also reduce the influence of solar irradiance, canopy structure, soil background, and other factors [51]. According to the vegetation indices used in previous studies on crop agronomic parameters, 15 commonly used vegetation indices were calculated from RGB images (Table 1), and 18 vegetation indices were calculated from multispectral images (Table 2).

### 2.4. Modeling Methods

A variety of feature variables extracted from UAV-based images were used as input variables to construct the estimation models of LAI, FW, and DW. Modeling methods included base machine learning model and ensemble learning model. The former included ridge regression (RR), SVM, random forest (RF), Gaussian process (GP), and K-neighbor network (K-NN). The uncertainty of the prediction results caused by the model structure and parameters may lead to the fact that the results of a base model may not well represent the relationship between the variables [79]. Compared with the individual models, the ensemble learning model can comprehensively consider the performance of each model and obtain more reliable results [80]. Therefore, two ensemble learning methods, stacked generalization and BMA, were used to compare with the basic models to improve the accuracy and reliability of LAI, FW, and DW estimation. The RR, SVR, RF, GPR, and K-NN were used as the basic models for ensemble learning.

Stacked generalization was put forward by Breiman [36], which is the generalization of multiple layers and models into a new model. Simple stacking generally includes primary and secondary models. The primary model is trained based on the original data, and then the output of the primary model is applied to the secondary model as a new input. In order to avoid the data overfitting, the training set is usually divided into *k* parts, and the cross-validation is used to train each model [10, 32, 33]. In general, the stacking model outperform than that of the basic model.

BMA is a special case of stacked generalization, which uses the posterior weights instead of multiple linear regression (MLR) to combine predictions of basic learners. BMA combines the Bayesian theory with model averaging, and the final model is obtained by a posteriori probability weighted averaging based on the model mathematical structure and all unknown parameters [81, 82]. BMA considers the uncertainty caused by model selection, including parameter uncertainty and model uncertainty. BMA use Bayesian theorem to obtain the model parameter and the posterior distribution of the model itself, can not only solve the problem of singularity model, but also directly select the model [83].

In this study, five machine learning methods superimposed on a two-layer model were used to estimate the LAI, FW, and DW of breeding maize based on UAV-based features. All the models were verified by 5-fold cross-validation.

**Figure 4:**
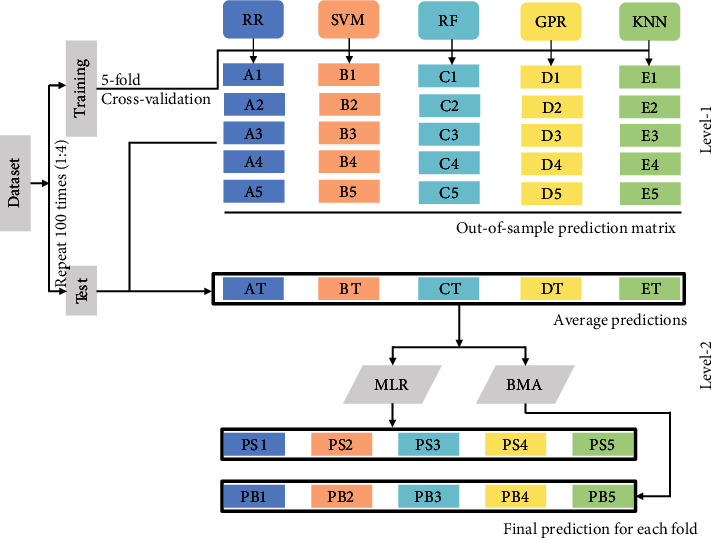
The workflow of the ensemble learning for maize traits estimation.

The estimation models of RR, SVR, RF, GPR, and KNN were first constructed, respectively, and then the prediction results were used as input variables to train and verify in the secondary layer using MLR and BMA. Finally, the estimation results of LAI, FW, and DW were obtained. The flow of ensemble learning is shown in Figure 4.

### 2.5. Model Performance Evaluation

A total of 220 samples were obtained at the four growth stages. 75% of the samples were used as the training set to construct the model, and the remaining 25% were used as the testing set to evaluate the model accuracy. In order to eliminate the random error, the modeling process was repeated for 100 times. The average result of the 100 repetitions was taken as the final result. The model evaluation indices include the determination coefficient (*R*^2^) and root mean square error (RMSE).

## 3. Results

### 3.1. Statistical Description of Phenotypic Traits

The statistical results of the measured PH, LAI, FW, and DW are shown in Table 3. There were five statistical indicators, including mean, maximum (Max), minimum (Min), standard deviation (SD), and coefficient of variation (CV). The dispersion degree was large for each phenotypic trait, and the CV was more than 50%, indicating that the plant line and growth stage had a great influence on the canopy structure. The large data span also provided the basis for the robustness of the model.

### 3.2. Plant Height Estimation

**Figure 5:**
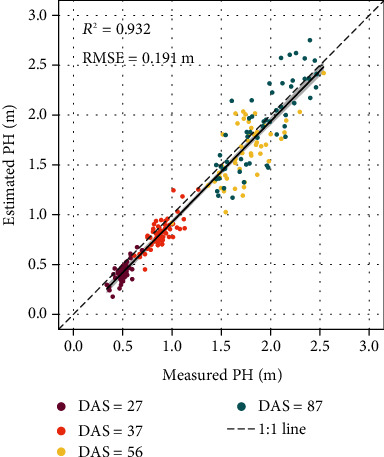
Scatter plot of the measured against estimated maize plant height.

**Figure 6:**
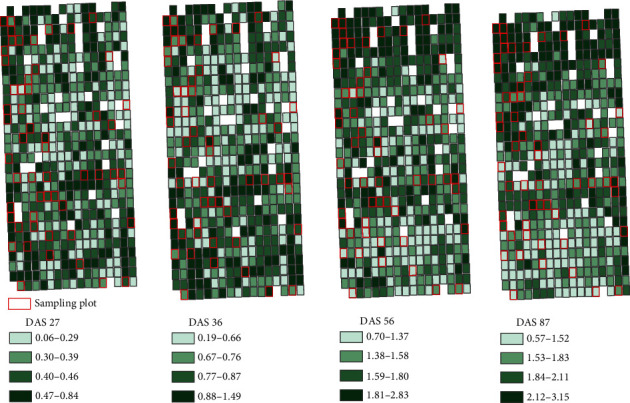
The heat map of estimated plant height.

For the sample data of four growth stages, the *R*^2^ and RMSE range of measured and estimated PH was 0.509~0.694 and 0.109~0.250 m (Figure 5). At the first three stages, there was a slight PH underestimation. At the latter stages, the measured and estimated PH had good consistency. During the whole growth stages, the *R*^2^ and RMSE of measured and estimated PH was 0.932 and 0.191 m, respectively, indicating that the maize PH based on RGB images had high estimation accuracy and could be used for the subsequent studies of LAI, FW, and DW.  Figure 6 is the heat map of estimated plant height.

### 3.3. Correlation between Feature Variables and Phenotypic Traits

**Figure 7:**
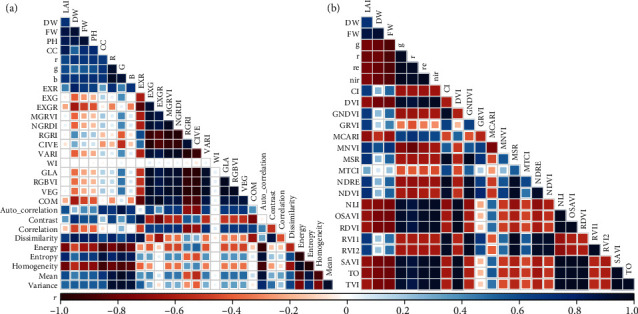
Correlation coefficient between maize phenotyping traits and feature variables from UAV-based RGB (a) and multispectral images (b).

In order to explore the correlation between different feature variables and LAI, FW, and DW, Pearson correlation analysis were conducted between UAV image features and measured phenotypic traits (Figure 7). PH and canopy coverage were highly correlated with phenotypic traits (Figure 7(a)). The correlation coefficients between PH and LAI, FW, and DW were 0.845, 0.866, and 0.928, respectively, indicating that structural parameters had great potential in estimating crop phenotype. The texture information was also strongly correlated with phenotypic traits (Figure 7(b)). The correlation between RGB spectral vegetation indices and phenotypic traits was the worst, especially LAI. Most RGB spectral vegetation indices were weakly correlated with LAI.

### 3.4. Validation of Phenotypic Traits

Tables 4–6 show the mean values of *R*^2^ and RMSE of LAI, FW, and DW models using all modeling methods in this study. Single-type feature variable combined with a base model could effectively estimate LAI, FW, and DW. The estimation accuracy was relatively close constructed with each base model. The model performance was slightly different due to different kinds of feature variables and phenotypic traits, among which RR and RF performed relatively better than the other three. Among the three kinds of features variables extracted from RGB images, the order of estimation accuracy was structural traits > texture > spectrum. In terms of five base models, the mean values of *R*^2^ of LAI, FW, and DW of the optimal estimation models constructed by RGB structural parameters were 0.819, 0.859, and 0.858, respectively, for the validation dataset. The estimation with multispectral vegetation indices was much higher than that with the vegetation indices from visible light bands. For the validation dataset, *R*^2^ of LAI, FW, and DW estimation increased by 55.680%, 32.663%, and 27.209%, respectively.

In order to compare the model performance before and after feature fusion, we analyzed the estimation accuracy of LAI, FW, and DW constructed by each basic modeling method. After the fusion of different feature variables, the estimation accuracy of various phenotypic traits was improved on the whole. For the RGB data, the model constructed using all feature variables simultaneously had the highest accuracy. As to the validation dataset, the mean values of *R*^2^ of LAI, FW, and DW model were 0.821, 0.871, and 0.864, respectively. It showed that feature fusion for different variables could improve the model estimation accuracy. On the basis of using three kinds of feature variables derived from RGB images, we added the multispectral features to construct estimation model of various phenotypic traits. According to the optimal model, the estimation accuracy of FW and DW based on the two sensors was improved to a certain extent compared with the RGB or multispectral sensor. For the validation dataset of five basic models with multisensor features, *R*^2^ of LAI, FW, and DW of the optimal estimation models were 0.836, 0.876, and 0.919, respectively. It indicated that multisensor data fusion could enhance the estimation accuracy and universality of the model. The optimal uncertainty estimates of three traits using GPR were shown in Supplement table 1-table 3.

**Figure 8:**
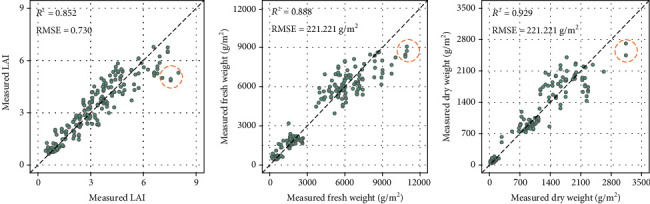
Scatter plot of the measured against optimal estimated phenotypic traits by BMA model in maize using validation dataset within 100 times.

The stacking and BMA models were used to further estimate the phenotypic traits by integrating the results of five base models. Regardless of multifeature variables or multisensor data fusion, the ensemble learning models performed better than the five basic models. Based on the ranking criteria of *R*^2^, the validation results of the optimal models for LAI, FW, and DW were 0.852, 0.887, and 0.929, respectively. The accuracy of ensemble learning model was slightly lower than that of RR when only structural parameters were used to estimate LAI. Although the ensemble learning model does not always performed best, it can minimize the deviation and randomness of the base model and make the model more stable. Therefore, the ensemble learning model further improved the generalization by combining the advantages of each basic model.  Figure 8 shows the scatter plot of the measured DW, LAI, and FW against the estimated values with BMA model using validation dataset. A good estimation result was achieved for each phenotypic trait. However, there were still slight underestimations of phenotypic traits at the later growth stage of maize.

### 3.5. Mapping Maize Phenotypic Traits

**Figure 9:**
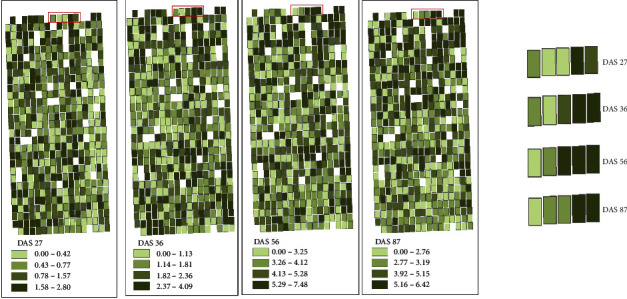
Estimation map of LAI using BMA-based multisensor data fusion.

**Figure 10:**
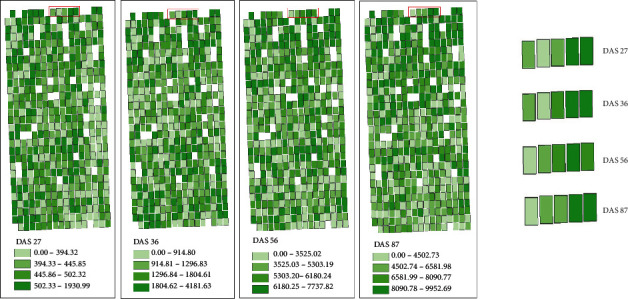
Estimation map of fresh weight using BMA-based multisensor data fusion.

**Figure 11:**
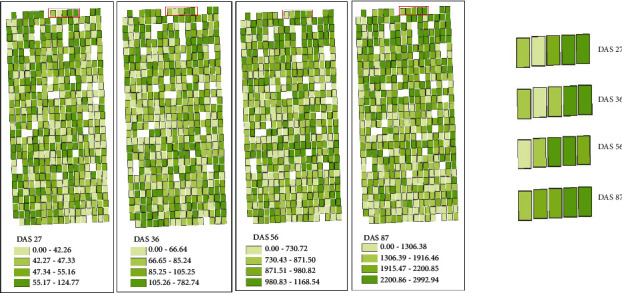
Estimation map of dry weight using BMA-based multisensor data fusion.

The LAI, FW, and DW of breeding maize at four growth stages were estimated and mapped using BMA estimation model constructed based on feature variables obtained from two kinds of images. Figures 9–11 show the LAI, FW, and DW among maize lines at each growth stage and their dynamic changes of each plot. The range of the classes for each variable (LAI, FW, and DW) was based on the quantile method in ArcGIS software. The LAI showed similar spatial distribution at each stage, indicating that different maize lines had consistent growth rate. It may be closely related to the genetic characteristics of the maize lines. In addition, the LAI distribution was consistent with PH, FW, and DW. On the whole, the plots with higher PH and LAI had higher FW and DW. The FW and DW of maize lines in the single stage were different, which may be caused by the adaptability of different maize lines to the local environment. For example, the life cycle of tropical maize lines would lengthen in the warm temperate continental monsoon climate.

## 4. Discussion

The maize PH was estimated using the UAV-based RGB images and validated with the measured values in this study. Good accuracy was achieved, and the *R*^2^ was 0.9 between the measured and estimated PH. Four kinds of feature variables (spectrum, texture, structure, and vegetation indices) were extracted from the digital images or multispectral images. Five basic models and two ensemble learning models were adopted in the modeling method. For LAI, FW, and DW, the fusion of multiple features could improve the estimation accuracy, and the ensemble learning models further improved the accuracy. High accuracy was realized to estimate the phenotypic traits of breeding maize by integrating multisource data fusion and ensemble learning.

The spectrum, texture, and structure information of UAV-based image have been widely used in crop phenotyping research [84–86]. The multispectral vegetation indices showed strong correlation with phenotypic traits. This is because multispectral images have richer spectral bands than RGB images, especially in the near infrared band, which is helpful to improve the correlation between maize phenotypic traits and vegetation indices. Similar to previous studies, spectrum data can well estimate LAI, FW, and DW here. The structure parameters such as plant height and canopy coverage also achieved high precision, indicating the great potential in crop phenotypic extraction and application. However, the single data source may have limitations, such as the spectrum saturation in the later stage of crop growth [12, 13, 87, 88]. To effectively solve the problem of spectrum saturation in the middle and later stage of maize, we tried to fuse different feature variables to improve the accuracy and universality of the model [4, 48, 89, 90]. Spectral vegetation indices were a kind of parameter commonly used in estimation of aboveground biomass and LAI of crops [26, 27, 91]. In previous studies, spectrum was used to estimate crop phenotypic traits alone, and the model combined with plant height, canopy coverage, and texture information achieved more accurate estimation [18, 92–96]. Similar results were also found in this study. Among the spectrum, structure, and texture information, the structural parameters had the best performance. The structural parameters + texture or structural parameters + spectrum can improve the model precision, among which the structural parameters + texture + spectrum performed the best. Similarly, multisensor data fusion can help to improve the accuracy of estimating phenotypic traits [97–99]. For example, compared with using single-type data source, combination of spectrum and thermal infrared data can increase the overall estimation precision of the model [100, 101]. Different from wheat aboveground biomass estimation by using expensive UAV hyperspectral data [18], good accuracy was also achieved, and the cost of data acquisition were greatly saved for different types of feature variables obtained from digital and multispectral images used in this study to estimate LAI, FW, and DW of breeding maize.

Crop growth is influenced by variety, field management, and environment. The phenotypic traits have complicated relationships with spectrum, structural parameter, and texture information. The conventional linear regression modeling may be difficult to express their relationships. With the rapid development of data mining, artificial intelligence, and crop phenotyping, phenotypic research based on machine learning has become a hot topic [102, 103]. Compared with the traditional linear regression, machine learning can achieve classification or regression with high precision through self-learning [104, 105]. The machine learning methods commonly used in crop phenotypic study include RF, SVM, and artificial neural network [92, 106]. RF method generally performed better than other methods in estimating phenotypic traits by statistical regression [25, 45, 107]. As to the five base models used in this study, satisfactory results were obtained in estimating LAI, FW, and DW of breeding maize, among which RF and RR had better performance than the others. Improving the accuracy and reliability of phenotypic acquisition is a prerequisite for selecting excellent genotypes. The model integration can combine the advantages of multiple base models and has higher estimation accuracy, robustness, and overall induction ability [108–111]. Feng et al. [32, 33] predicted alfalfa yield using UAV-based hyperspectral data and found that the accuracy of the integrated model was superior to all basic models. Due to the practical limitations, we obtained the phenotypic traits of 55 sample plots at each growth stage. Compared with the large sample set, the output of various model may have great differences. Ensemble learning can provide a unified and consistent model through decision-level fusion. Therefore, taken five machine learning methods as basic models, the ensemble learning methods, included stacking and BMA, were used to improve the accuracy and reliability of maize phenotypic traits estimation. The results showed that both stacking and BMA performed better than the basic modeling methods in estimating the LAI, FW, and DW of breeding maize.

Our results showed that the fusion of multisource data combined with model ensemble learning method can estimate the LAI, FW, and DW of breeding maize with high accuracy. The study could provide significant guidance for UAV imaging technology to study crop phenotypes. In this study, only three phenotypic parameters were studied. The data fusion and model integration could be applied to more breeding phenotypic traits in the future, such as crop biochemical parameters, nitrogen content, chlorophyll content, and protein content. In addition, thermal infrared imaging can be used to obtain crop canopy temperature, which is widely used to monitor water stress, freezing stress, and yield estimation [94, 112, 113]. We will add thermal infrared data to further explore its ability in the estimation of breeding phenotypic traits in the follow-up study. Compared with conventional machine learning methods, deep learning can better mine the potential of data and greatly improve the research accuracy in many aspects [114, 115]. In the following studies, we will try to introduce the combination of deep learning and ensemble learning to further explore the application ability of UAV-based imaging technology in breeding maize phenotypes.

## 5. Conclusion

This study evaluated the contribution of different feature variables from RGB sensor, feature variable of same type from different sensors, and fusion data to LAI, FW, and DW of breeding maize. The integrated model framework was built based on five machine learning methods, including stacking and BMA, to estimate LAI, FW, and DW of maize. The results showed that no matter which modeling methods, the performance of multisource data fusion was better than that of single kind of feature variables on estimating LAI, FW, and DW. Among the five single machine learning methods, RF and RR performed better than the other three. Both stacking and BMA model improved the estimation accuracy compared to each machine learning method. After all data of the two sensors were fused, for the LAI, FW, and DW, the of the ensemble learning model increased by 1.088%-5.448%, 1.37%-11.854%, and 1.914%-12.698%, respectively, compared with those of the basic models. The data fusion of UAV digital and multispectral sensors improved the estimation accuracy, while the ensemble learning model further improved the estimation accuracy of phenotypic traits. In this study, multisource data fusion and ensemble learning model were combined to realize high-accuracy estimation of LAI, FW, and DW of breeding maize, which could provide support for high-throughput extraction of phenotypic traits in crop breeding.

## Figures and Tables

**Table 1:** Vegetation index calculation formula of RGB images.

Vegetation indices	Definition	References
*g*, *r*, *b*	The DN value of each band	/
EXR	1.4∗*r* − *g*	[52]
EXG	2∗*g* − *r* − *b*	[53]
EXGR	3∗*g* − 2.4∗*r* − *b*	[54]
MGRVI	(*g*^2^ − *r*^2^)/(*g*^2^ + *r*^2^)	[4]
NGRDI	(*g* − *r*)/(*g* + *r*)	[55]
RGRI	*r*/*g*	[56]
CIVE	0.441∗*r* − 0.881∗*g* + 0.385∗*b* + 18.78	[57]
VARI	(*g* − *r*)/(*g* + *r* − *b*)	[58]
WI	(*g* − *b*)/(*r* − *g*)	[53]
GLA	(2∗*g* − *r* − *b*)/(2∗*g* + *r* + *b*)	[59]
RGBVI	(*g*^2^ − *b*∗*r*)/(*g*^2^ + *b*∗*r*)	[60]
VEG	*g*/(*r*^*k*^ *b*^1−*k*^), *k* = 0.667	[61]
COM	0.25∗EXG + 0.3∗EXGR + 0.33∗CIVE + 0.12∗VEG	[59]

Note: g: green; r: red; b: blue.

**Table 2:** Vegetation index calculation formula for multispectral images.

Vegetation indices	Definition	References
*g*, *r*, re, nir	The DN value of each band	/
CI	(nir/re) - 1	[62]
DVI	nir - *r*	[63]
GNDVI	(nir-g)/(nir+g)	[64]
GRVI	(*g*-*r*)/(*g*+*r*)	[61]
MCARI	((re − *r*) − 0.2∗(re − *g*))∗(re/*r*)	[65]
MNVI	(1.5∗(nir^2^ − *r*))/(nir^2^ + *r* + 0.5)	[66]
MSR	(nir/*r*-1)/(sqrt(nir/*r*)+1)	[67]
MTCI	(nir-re)/(re-*r*)	[68]
NDRE	(nir-re)/(nir+re)	[69]
NDVI	(nir-*r*)/(nir+*r*)	[70]
NLI	(nir^2^-*r*)/(nir^2^+*r*)	[71]
OSAVI	(1.16∗(nir − *r*)/(nir + *r* + 0.16))	[72]
RDVI	(nir-*r*)/(sqrt(nir+*r*))	[73]
RVI1	nir/*r*	[74]
RVI2	nir/*g*	[75]
SAVI	1.5∗(nir − *r*)/(nir + *r* + 0.5)	[76]
TO	3∗((reg − *r*) − 0.2∗(reg − *g*)∗(reg/*r*))/OSAVI	[77]
TVI	60∗(nir − *g*) − 100∗(*r* − *g*)	[78]

Note: g: green; r: red; re: red-edge; nir: near-infrared.

**Table 3:** Statistics of the measured phenotypic traits.

Variables	Mean	Min	Max	SD	CV
PH (m)	1.265	0.338	2.538	0.627	49.510%
LAI	3.068	0.382	9.636	1.876	61.159%
FW (g/m^2^)	3710.752	160.448	12204	2919.349	108.182%
DW (g/m^2^)	739.532	18.7	3699.765	802.259	78.673%

Note: CV: coefficient of variation; SD: standard deviation.

**Table 4:** Validation of different models for LAI estimation.

Sensor type	Feature type	Variables num	Metrics	RR	SVM	RF	GPR	KNN	Stacking	BMA
RGB	Spe	16	*R* ^2^	0.537	0.521	0.522	0.536	0.521	0.567	0.567
			RMSE	1.303	1.330	1.309	1.285	1.316	1.244	1.244
	Str	2	*R* ^2^	0.819	0.773	0.787	0.793	0.805	0.816	0.817
			RMSE	0.808	0.911	0.875	0.868	0.836	0.815	0.810
	Tex	9	*R* ^2^	0.770	0.718	0.718	0.727	0.719	0.775	0.775
			RMSE	0.912	1.022	1.007	1.000	1.008	0.902	0.900
	Spe + Str	18	*R* ^2^	0.837	0.750	0.807	0.749	0.727	0.837	0.840
			RMSE	0.765	0.955	0.832	0.956	0.994	0.762	0.756
	Spe + Tex	25	*R* ^2^	0.765	0.719	0.741	0.723	0.718	0.781	0.781
			RMSE	0.924	1.019	0.964	1.006	1.009	0.886	0.888
	Str + Tex	11	*R* ^2^	0.817	0.778	0.794	0.778	0.765	0.818	0.822
			RMSE	0.815	0.902	0.860	0.905	0.919	0.812	0.801
	Spe + Str + Tex	27	*R* ^2^	0.821	0.758	0.807	0.756	0.743	0.832	0.835
			RMSE	0.809	0.941	0.834	0.946	0.964	0.780	0.772
MS	Spe	22	*R* ^2^	0.836	0.787	0.824	0.785	0.791	0.841	0.842
			RMSE	0.767	0.884	0.795	0.885	0.869	0.755	0.751
RGB + MS	Spe + Str + Tex	49	*R* ^2^	0.817	0.763	0.836	0.760	0.756	0.852	0.852
			RMSE	0.824	0.931	0.768	0.933	0.939	0.730	0.730

Note: Spe: spectral features; Str: structure features; Tex: texture features.

**Table 5:** Validation statistics of different models for fresh weight (g/m^2^) estimation.

Sensor	Feature type	Variables num	Metrics	RR	SVM	RF	GPR	KNN	Stacking	BMA
RGB	Spe	16	*R* ^2^	0.639	0.626	0.646	0.624	0.639	0.663	0.665
			RMSE	1782.5	1828.8	1754.4	1805.6	1772.2	1711.6	1704.8
	Str	2	*R* ^2^	0.859	0.818	0.849	0.831	0.846	0.859	0.861
			RMSE	1100.4	1266.0	1140.1	1232.4	1147.7	1103.1	1095.0
	Tex	9	*R* ^2^	0.787	0.761	0.764	0.759	0.775	0.803	0.803
			RMSE	1364.1	1467.8	1434.8	1461.4	1406.0	1311.7	1307.5
	Spe + Str	18	*R* ^2^	0.866	0.784	0.851	0.786	0.799	0.87	0.873
			RMSE	1077.0	1376.1	1133.6	1376.8	1323.9	1063.7	1046.7
	Spe + Tex	25	*R* ^2^	0.766	0.743	0.761	0.749	0.778	0.797	0.799
			RMSE	1437.5	1513.6	1446.0	1491.1	1395.9	1332.1	1323.1
	Str + Tex	11	*R* ^2^	0.866	0.805	0.846	0.805	0.804	0.865	0.868
			RMSE	1079.9	1313.9	1151.0	1321.0	1304.7	1084.8	1065.9
	Spe + Str + Tex	27	*R* ^2^	0.871	0.778	0.849	0.784	0.8	0.877	0.879
			RMSE	1058.5	1394.2	1140.0	1386.2	1320.9	1035.8	1022.4
MS	Spe	22	*R* ^2^	0.857	0.849	0.856	0.846	0.838	0.865	0.865
			RMSE	1121.8	1176.0	1124.7	1173.4	1203.2	1088.7	1088.5
RGB + MS	Spe + Str + Tex	49	*R* ^2^	0.858	0.793	0.876	0.799	0.823	0.888	0.887
			RMSE	1118.6	1348.0	1035.9	1332.3	1242.2	988.9	987.3

Note: Spe: spectral features; Str: structure features; Tex: texture features.

**Table 6:** Validation statistics of different models for dry weight (g/m^2^) estimation.

Sensor type	Feature type	Variables num	Metrics	RR	SVM	RF	GPR	KNN	Stacking	BMA
RGB	Spe	16	*R* ^2^	0.713	0.669	0.71	0.676	0.693	0.723	0.727
			RMSE	442.3	474.9	441.8	468.0	454.1	432.4	427.6
	Str	2	*R* ^2^	0.814	0.821	0.858	0.832	0.849	0.862	0.865
			RMSE	352.3	348.6	304.7	346.4	316.6	302.7	299.6
	Tex	9	*R* ^2^	0.766	0.768	0.768	0.761	0.777	0.798	0.802
			RMSE	396.8	401.8	396.9	405.8	388.8	369.3	365.3
	Spe + Str	18	*R* ^2^	0.846	0.789	0.861	0.788	0.803	0.864	0.869
			RMSE	321.1	377.9	304.6	382.8	363.3	301.7	296.1
	Spe + Tex	25	*R* ^2^	0.745	0.737	0.77	0.738	0.767	0.781	0.788
			RMSE	418.4	424.0	394.6	423.0	396.7	384.5	377.8
	Str + Tex	11	*R* ^2^	0.853	0.831	0.865	0.827	0.829	0.869	0.872
			RMSE	315.6	339.7	300.3	350.0	339.5	296.6	293.4
	Spe + Str + Tex	27	*R* ^2^	0.853	0.789	0.864	0.786	0.798	0.875	0.879
			RMSE	314.5	377.0	302.0	385.4	368.7	289.7	284.8
MS	Spe	22	*R* ^2^	0.907	0.906	0.905	0.901	0.887	0.914	0.913
			RMSE	253.3	260.8	256.7	266.6	283.1	245.2	246.1
RGB + MS	Spe + Str + Tex	49	*R* ^2^	0.898	0.851	0.919	0.849	0.881	0.929	0.929
			RMSE	264.4	318.7	236.3	324.7	286.2	221.6	221.2

Note: Spe: spectral features; Str: structure features; Tex: texture features.

## Data Availability

The data used in this study are freely available. Anyone who wants to use the data can contact the corresponding author Yuntao Ma. The author is with the College of Land Science and Technology, China Agricultural University, Beijing, 100193, China (e-mail: yuntao.ma@cau.edu.cn).

## Supplementary Materials

Supplementary Materials The optimal uncertainty estimates of three traits using GPR were shown in Supplement table 1-table 3.
